# High-Quality and High-Efficiency Fabrication of Microlens Array by Rotary Profile Cutting Method

**DOI:** 10.3390/mi16121374

**Published:** 2025-12-01

**Authors:** Liheng Gao, Xiuwen Sun, Qian Yu, Yinhui Wang, Md Nasir Uddin, Ruijue Duan, Gang Wang, Zhikang Zhou, Qiuchen Xie, Tao Sun, Tianfeng Zhou

**Affiliations:** 1 State Key Laboratory of Chips and Systems for Advanced Light Field Display, Beijing Institute of Technology, Beijing 100081, China; 2 School of Mechanical Engineering, Beijing Institute of Technology, Beijing 100081, China; 3 Department of Mechanical and Energy Engineering, Southern University of Science and Technology, Shenzhen 518055, China; 4 School of Medical Technology, Beijing Institute of Technology, Beijing 100081, China; 5 Chongqing Innovation Center, Beijing Institute of Technology, Chongqing 401120, China

**Keywords:** microlens array, rotary profile-cutting, tool setting errors, ultraprecision machining, glass molding

## Abstract

To enhance the fabrication consistency and surface quality of microlens array (MLA) molds, this study presents a high-quality and high-efficiency rotary profile-cutting (RPC) method conducted on a four-axis ultraprecision machining platform. A geometric model is established to define the relationship between tool parameters and microlens structural features, and the toolpath is optimized by refining control points to enhance machining accuracy. In addition, a novel tool-setting error characterization approach is developed, enabling submicron-level positioning of the diamond tool, with errors in the X and Y directions controlled within 1 μm. Experimental validation demonstrates the successful fabrication of a 6 × 6 square-array MLA mold with a curvature radius of 507 μm using the proposed RPC method. Subsequent replication of MLA through precision glass molding (PGM) yielded structures with a peak-to-valley (PV) value below 354 nm and surface roughness (Ra) below 11 nm. Optical performance tests confirm the high consistency and accuracy of the fabricated MLA, highlighting the potential of the proposed RPC technique for advanced optical component manufacturing.

## 1. Introduction

Microlens arrays (MLAs) are miniature optical components consisting of an array of microlens units with apertures ranging from 1 μm to 1 mm. These units exhibit specific shapes and arrangements, offering advantages such as compact size and high integration density [1,2]. These unique characteristics enable MLA to perform a variety of optical functions, including beam homogenization, spot shaping, and aberration correction [3,4]. With the increasing demand for device miniaturization and integration, MLA have become indispensable in a wide range of high-tech civilian and defense applications, including communications, light-field cameras, displays, and artificial intelligence [5,6]. However, the complex periodic structure layouts, and stringent requirements for contour accuracy pose significant challenges to the high-quality fabrication of MLA [7].

Various techniques, including laser direct writing [8], photolithography [9], micro-electrical discharge machining [10], injection molding [11], hot melt reflow [12], and chemical vapor deposition [13], have been employed for the fabrication of MLA. While these methods are versatile, their reliance on high-cost equipment, complex processing steps, and material limitations restricts their widespread adoption. In contrast, utilizing diamond tools for ultraprecision mold machining, combined with replication techniques for the mass production of optical components, provides a more practical solution for large-scale MLA fabrication [14,15].

Currently, ultraprecision machining methods for MLA molds typically include slow tool servo (STS) turning [16], fast tool servo (FTS) turning [17], micro-milling [18], and direct laser writing lithography [19]. Sun et al. [20] employed STS turning with a novel surface morphology prediction model for MLA, applying Gaussian filtering to improve machining quality, precision, and efficiency. Despite these advances, tool rotation around the microlens center combined with tool-setting errors induced distortions in the MLA surface profile. Kong et al. [21] developed a deterministic model for generating MLA surfaces using the FTS turning method, integrating cutting mechanics, tool geometry, machining parameters, and surface profile analysis to accurately predict surface quality. However, it remained affected by hysteresis effects in the FTS system and tool wear. Kim et al. [22] introduced a tilted micro-milling method to address surface defects in MLA caused by tool errors in traditional milling processes, significantly improving machining accuracy and surface quality through tool axis adjustment. Nevertheless, the required multi-axis motion for spiral toolpath led to cumulative motion errors that reduce the machining consistency. Malinauskas et al. [23] employed ultrafast laser 3D writing followed by calcination to produce high-quality, free-form glass-ceramic microlenses with excellent optical performance and environmental stability. Yet the additive writing process and the high-temperature calcination step make it difficult to achieve high throughput and large-area uniformity, limiting its applicability in MLA fabrication.

Four-axis machining platform, characterized by their structural simplicity and ease of control, offer effective reduction in error accumulation during complex multi-axis operations. They offer high machining accuracy along with improved precision and stability, making them particularly well-suited for MLA mold machining, especially in small- to medium-batch production with stringent precision requirements. In this study, a rotary profile-cutting (RPC) method is proposed for fabricating MLA mold, based on a four-axis horizontal ultraprecision machining platform comprising X-, Z-, C-, and B-axes. The method employs a diamond tool mounted such that its rake face is parallel to the Y-Z plane. Machining of each MLA unit is initiated by the coordinated rotation of the C-axis and servo feeding of the X- and Z-axes, enabling accurate material removal through profile-cutting. Compared with existing ultraprecision machining approaches that often suffer from dynamic lag, cumulative motion errors, or limited surface controllability, the proposed RPC method leverages a deterministic profile-cutting strategy and a stable four-axis configuration to achieve highly consistent and precise MLA mold fabrication.

While this unconventional tool mounting configuration offers advantages, it also imposes strict requirements on tool-setting accuracy. Specifically, positional errors of the diamond tool in the X and Y directions have a significant impact on the machining accuracy of MLA structures. Conventional tool-setting methods, which typically depend on analyzing residual errors after machining, are inadequate for accurately detecting and characterizing these critical deviations. Consequently, the development of a precise and reliable method for tool-setting error characterization is essential to ensure the high-quality fabrication of MLA molds.

To address these challenges, this study systematically investigates the relationship between tool parameters and the structural characteristics of MLA molds in the RPC process. Based on this analysis, precision tool motion trajectories were generated to optimize the machining strategy. Furthermore, a rapid characterization method was also developed to accurately quantify tool-setting errors in both the X and Y directions. To validate the proposed approach, a 6 × 6 MLA mold was fabricated and subsequently replicated on MLA lenses using precision glass molding (PGM) technology, which does not introduce any noticeable changes to the optical transmission properties of the glass. The quality of the fabricated components was evaluated by analyzing measurements of three-dimensional morphology, profile accuracy, and surface roughness of the MLA lenses. In addition, an optical imaging platform was developed to evaluate the optical performance of the fabricated lenses.

## 2. Methods

### 2.1. Basic Principles of RPC for MLA Mold Fabrication

**Figure 1:**
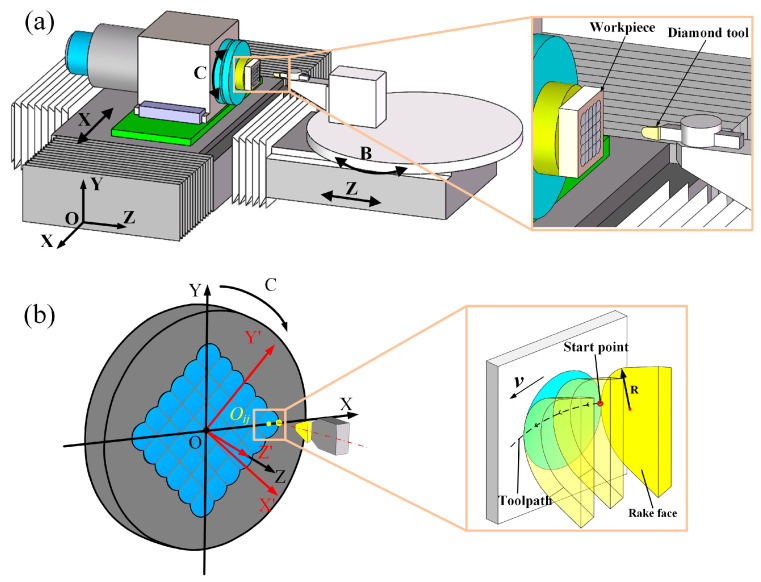
Principle of RPC machining process: (**a**) four-axis machining platform; (**b**) material removal mechanism for microlens generation.

The RPC method is implemented on a four-axis horizontal ultraprecision machining platform, as illustrated in Figure 1a. In this configuration, the X- and Z-axes provide precise linear motions, while the C- and B-axes enable controlled rotational movements. The workpiece is securely mounted on the C-axis using a vacuum chuck, ensuring stable positioning during machining. A single-point diamond tool is rigidly fixed on the B-axis via a precision tool holder, with its rake face aligned parallel to the Y-Z plane to maintain optimal cutting orientation.

The ultraprecision machining process of the MLA using the RPC method is illustrated in Figure 1b. The center of the C-axis is defined as the origin O, establishing both the machine coordinate system (O-XYZ) and the workpiece coordinate system (O-X′Y′Z′). The toolpath is programmed to rotate around the geometrical center of the MLA at O. The center coordinates *O_ij_* of each microlens are determined based on the MLA design. To initiate machining, the C-axis rotates to align the selected microlens center *O_ij_* with the X-axis, and then the X-axis moves linearly to position the diamond tool at the starting point of each microlens. Material removal is subsequently carried out via synchronized interpolation of the X- and Z-axes, where the microlens profile is defined by both the geometry of the diamond tool’s cutting edge and its motion trajectory. By repeating this cycle for each microlens unit, the entire MLA can be accurately fabricated.

**Figure 2:**
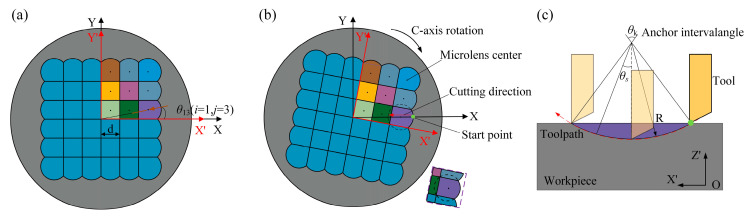
Machining program design for MLA mold fabrication: (**a**) schematic diagram of quadrant-based partition control; (**b**) generation of the microlens machining toolpath; (**c**) toolpath strategy for profile-cutting of microlens units.

The curvature radius of each microlens is primarily determined by the geometric profile of the cutting tool. Based on the preceding analysis, a 6 × 6 square-arrayed MLA structure was designed, as shown in Figure 2a. The positioning of individual microlens units was achieved through coordinated motion of the multi-axis machining operation, as shown in Figure 2b.

In the machining program design, a workpiece coordinate system is established with the center of the MLA defined as the origin. For simplification, only the first-quadrant region of the MLA is selected for analysis. The area is divided into a 3 × 3 grid, where the indices *i* and *j* range from 1 to 3. For square units with a side length of *d*, the distance between the center of any microlens and the coordinate origin can be expressed as:

(1) $$l_{ij}\;=\;\sqrt{{\left[\left(j\;-\;0.5\right)d\right]}^{2}\;+\;{\left[\left(i\;-\;0.5\right)d\right]}^{2}}$$

The angle between the X-axis and the vector from the origin to *O_ij_* is denoted as *θ_ij_*:

(2) $$\theta_{ij}=\arctan\left(\frac{\left(j-0.5\right)d}{\left(i-0.5\right)d}\right)=arctan\left(\frac{j-0.5}{i-0.5}\right)$$

The fabrication method proposed in this study enables each microlens to be machined in a single profile-cutting pass, as illustrated in Figure 2c. As a result, the initial positioning of the tool has a critical influence on the final forming accuracy of each microlens. The radial distance to the machining start points, defined in polar coordinates, is given by:

(3) $$\rho_{ij\;}=\;\sqrt{{\left[\left(j\;-\;0.5\right)d\right]}^{2}+{\left[\left(i\;-\;0.5\right)d\right]}^{2}}\;+\;\frac{\sqrt{2}}{2}d$$

The entire MLA is divided into four quadrants and represented as a periodic replication pattern derived from the first quadrant (*N* = 1), following the cyclic propagation rules described below:

(4) $$\theta_{N}\;=\;\theta_{ij}\;+\;\left(N\;-\;1\right)\;\frac{\pi }{2}$$

For the fabrication of each microlens, the toolpath is discretized into *T* angularly equidistant points, as illustrated in Figure 2c. The angular sampling positions for each microlens are defined as follows:

(5) $$\theta_{s}=\frac{\theta_{k}}{2}-(t-1)\frac{\theta_{k}}{\left(T-1\right)}$$

Here, *θ_s_* denotes the angle between the line connecting a discretized point to the microlens center and the microlens central axis. *θ_k_* represents the sector angle defined between the start and end point of the microlens unit with respect to its center, and *t* is the discretization index. Based on these geometric parameters, the corresponding machining positions in the workpiece coordinate system can be calculated using the validated computational method described as follows, where *x_ij_* and *z_ij_* represent the discrete coordinate points along the X- and Z-axis directions, respectively.

(6) $$\left\{\begin{array}{c} x_{ij}=R\left(\cos\frac{\theta_{k}}{2}-\cos\theta_{s}\right)\\ z_{ij}=R\sin\theta_{s}+l_{ij} \end{array}\right.$$

### 2.2. Effect of Tool Setting Errors During RPC

In the RPC machining of the MLA mold, the mold surface is first flattened through precision turning to accurately establish the relative position of the tool along the Z-axis. However, tool-setting errors in the X- and Y-axes directions can lead to a positional deviation of the microlens center from *O_ij_* to *O*′*_ij_*. This reduction in positioning accuracy adversely impacts the overall machining quality, particularly introducing significant positional errors in edge units of the MLA.

**Figure 3:**
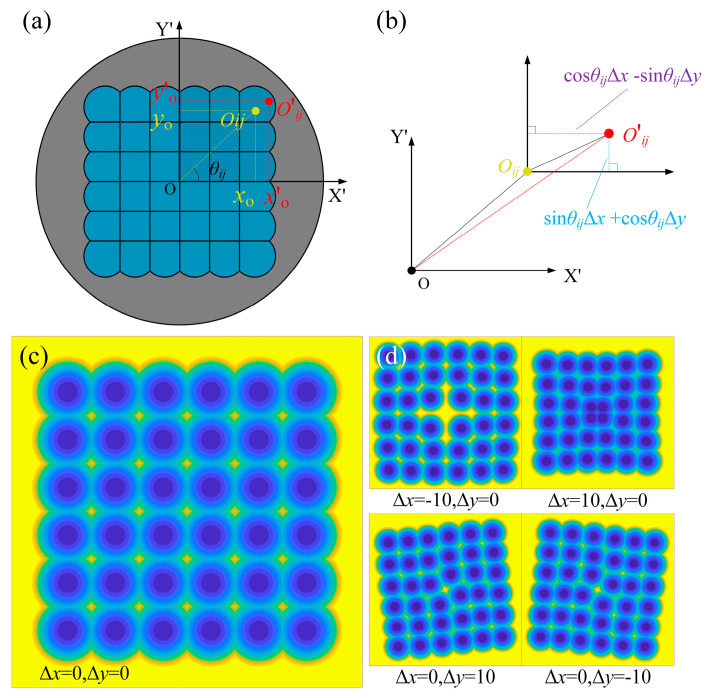
Influence of tool-setting errors on the of MLA fabrication: (**a**) deviation of the actual center caused by tool-setting errors actual; (**b**) calculation of the actual center offset; (**c**) MLA structure without tool-setting errors; (**d**) structural distortion of MLA induced by tool-setting errors.

As shown in Figure 3a,b, *Δx* and *Δy* represents the tool-setting errors in the X and Y directions, respectively. In the planar coordinate system, taking the microlens unit located in the upper-right corner as an example, the coordinates of its center point *O_ij_* can be expressed as follow:

(7) $$O_{ij}\;=\;\left[\begin{array}{c} x_{o}\\ y_{o} \end{array}\right]$$

The point *O_ij_* has coordinates *x_o_* and *y_o_* along the X and Y directions, respectively. These coordinates can be represented in either Cartesian or polar form, as follows:

(8) $$\left[\begin{array}{c} x_{o}\\ y_{o} \end{array}\right]\;=\;\left[\begin{array}{c} \left(i\;-\;0.5\right)d\\ \left(j\;-\;0.5\right)d \end{array}\right]\;=\;\left[\begin{array}{c} \rho_{o}\cos\theta_{ij}\\ \rho_{o}\sin\theta_{ij} \end{array}\right]$$

The shifted position *O*′ is represented by its coordinates *x*′*_o_* and *y*′*_o_*. The corresponding positional offset can be calculated using the following equations:

(9) $$O^{‘}\;=\;\left[\begin{array}{c} x_{o}^{‘}\\ y_{o}^{‘} \end{array}\right]\;=\;\left[\begin{array}{c} x_{o}+\cos\theta_{ij}\Delta x-\sin\theta_{ij}\Delta y\\ y_{o}+\sin\theta_{ij}\Delta x+\cos\theta_{ij}\Delta y \end{array}\right]$$

As shown in Figure 3c, the ideal arrangement of the MLA, without tool-setting errors in the X and Y directions, is presented. During the machining process, every four adjacent microlens converge at a common intersection point, which corresponds to a local highest maximum and is clearly visible in the resulting structure. From Equation (9), it can be observed that the tool-setting errors introduce nonlinear deviations in the position of each microlens center *O_ij_*. These deviations are strongly influenced by both the magnitude of the error and the angular position *O_ij_* in the polar coordinate system. Since *O_ij_* varies across different microlens positions within the array, the resulting positional errors are non-uniform, leading to spatial inconsistencies across the MLA.

As illustrated in Figure 3d, the influence of tool-setting errors *Δx* and *Δy* on MLA structure can be derived from Equation (9). When the error exists solely in the X-direction, the microlens units undergo uniform expansion or contraction relative to the array center. Specifically, *Δx* > 0 leads to outward expansion, resulting in an uncut region at the center of the MLA, whereas *Δx* < 0 causes inward contraction, leading to overcutting and interference at the center. In this case, the array retains symmetry along both the horizontal and vertical axes. In contrast, when the tool-setting errors occur exclusively in the Y-direction, the MLA units exhibit a rotational shift about the array center. For *Δy* > 0, the array undergoes a counterclockwise rotation, while for *Δy* < 0, it undergoes a clockwise rotation. This rotation entirely disrupts the array’s symmetry, with the displacement most pronounced at the central unit and increasing angularly with distance from the center. Since such positional errors degrade both the structural integrity and machining quality, it is essential to establish a reliable method for accurately measuring diamond tool-setting errors to ensure fabrication precision.

### 2.3. Control Method of Tool Setting Errors

In the actual machining operation, the toolpath exhibits a certain width *G_w_*, primarily determined by the tool radius *R* and cutting depth *C_d_*. The tool radius governs the lateral extent range of the toolpath, while the cutting depth influences its longitudinal geometric profile. An increase in either tool radius or cutting depth leads to a corresponding expansion in toolpath width. Moreover, the cutting depth serves as a critical parameter for calibrating toolpath width measurements. By fine-tuning the cutting depth, the actual width of the toolpath can be precisely controlled. Therefore, the toolpath width is governed by the combined effect of *R* and *C_d_*, with its geometric attributes offering a vital basis for evaluating machining precision and tool-setting errors. The interrelationship among these parameters can be summarized as follows:

(10) $$G_{w}\;=\;2\sqrt{R^{2}\;-\;{\left(R\;-\;C_{d}\right)}^{2}}$$

**Figure 4:**
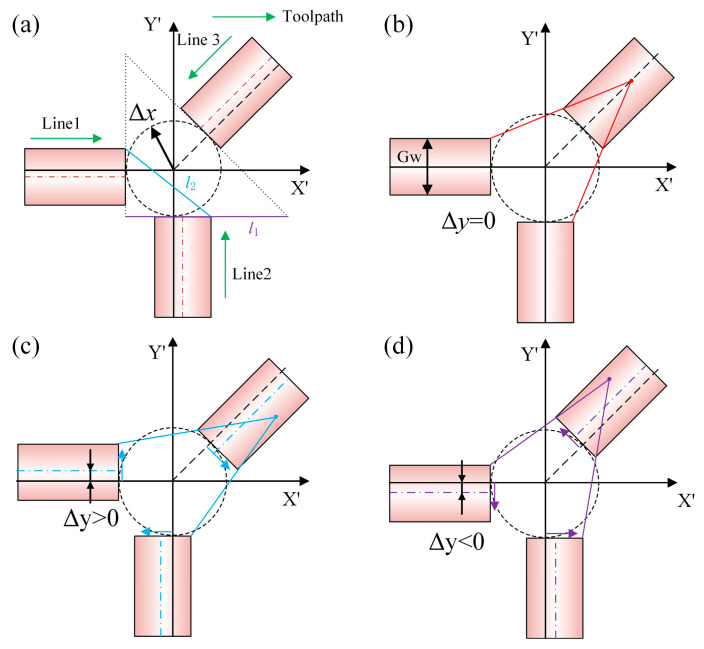
Tool-setting errors characterization method: (**a**) tri-line precision calibration approach; (**b**) *Δy* = 0; (**c**) *Δy* > 0; (**d**) *Δy* < 0.

During the tool-setting process, accurately measuring tool-setting errors after machining is particularly challenging due to the absence of a reference coordinate system on the machined surface. To overcome this issue, the present study analyzes the positional relationships among selected toolpaths to extract reliable reference information for tool-setting calibration. Three representative toolpaths are selected to investigate the effect of tool-setting errors. In the experimental procedure, the tool first moves from the edge toward the center along the positive X-axis to machine Line1. Then, the C-axis is rotated 90° clockwise to perform the machining of Line2. Finally, the C-axis is rotated to 225° to complete Line3, as illustrated in Figure 4a. In the RPC process, a tri-line tool-setting method is proposed based on the linear motion characteristics of the tool. In the absence of tool wear, burr formation, and tool-setting errors, the toolpaths generated by linear movements from the workpiece edge to the programmed origin point (0, 0) at different C-axis angles should theoretically converge at a single point. Line1, Line2, and Line3 represent toolpaths oriented at three distinct angular positions, and their ideal intersection is the machine’s rotational center. However, in the presence of tool-setting errors, these toolpaths fail to converge and instead form an intersecting pattern. This deviation from convergence can be exploited to characterize tool-setting errors, providing a critical foundation for subsequent error analysis and compensation.

In the preliminary analysis of tool-setting errors, the X-direction error is primarily reflected in the distance between the toolpath endpoint and the rotational center. Nevertheless, in actual characterization, repeated tool movements to the rotational center may cause machining interference and residual material buildup, complicating accurate measurement. To avoid overlapping tool marks, this study adopts a pre-set error method, in which the tool moves from the workpiece edge to a position offset from the rotational center by a fixed distance. This approach enables effective characterization of X-direction tool-setting errors. In contrast, Y-direction tool-setting errors manifest as an overall lateral shift in the toolpath. Ideally, the toolpath should pass through the C-axis rotational center; however, due to Y-direction errors, the actual path exhibits a systematic offset surrounding the rotational center. By analyzing the magnitude and pattern of this deviation, the tool-setting error in the Y-direction can be quantitatively determined.

Analysis of the X-direction tool-setting errors indicates that, in theory, the endpoints of the toolpaths should lie on a tangent circle with a radius equal to *Δx*. However, accurately determining the radius in practical measurements remains a significant challenge. To address this, the present study adopts a method based on the geometric extension of toolpath end lines. By extending three toolpaths, their intersection points form an isosceles right triangle. Using the geometric relationship between the triangle side length *l*_1_ and *Δx*, the calculation formula shown in Equation (11) is derived. For characterizing the Y-direction tool-setting errors, the geometric relationship between the tip distance *l*_2_ of Line1 and Line2 and the tool-setting errors *Δy* is analyzed. Based on this relationship, mathematical models are established, as shown in Equations (12) and (13), enabling the precise calculation of *Δy*.

(11) $$\Delta x=\frac{l_{1}}{\left(2+\sqrt{2}\right)}$$

(12) $${\left(\Delta x+\frac{G_{w}}{2}-\Delta y\right)}^{2}+{\left(\Delta x+\frac{G_{w}}{2}+\Delta y\right)}^{2}=l_{2}^{2}$$

(13) $$\Delta y=\pm \sqrt{\frac{l_{2}^{2}}{2}-{\left(\Delta x+\frac{G_{w}}{2}\right)}^{2}}$$

The preceding analysis enables the determination of the absolute value of *Δy*, but its sign, whether positive or negative, must be further identified based on the positional relationship of the tool tip. As illustrated in Figure 4b, when *Δy* = 0, the tool tip is precisely aligned with the C-axis rotation center. When the tool tip is positioned above the rotation axis, the extensions of the upper endpoints of Line1 and Line3, as well as those of the right endpoint of Line2 and the lower endpoint of Line3, intersect below the centerline of Line3. This configuration indicates *Δy* > 0, as shown in Figure 4c. Conversely, when the tool tip lies below the rotation axis, the same sets of extensions intersect above the centerline of Line3, corresponding to *Δy* < 0, as depicted in Figure 4d. By analyzing these relative geometric relationships, the sign and full value of *Δy* can be accurately determined.

## 3. Experimental Setup

### 3.1. Rotary Profile Cutting for MLA Mold

**Figure 5:**
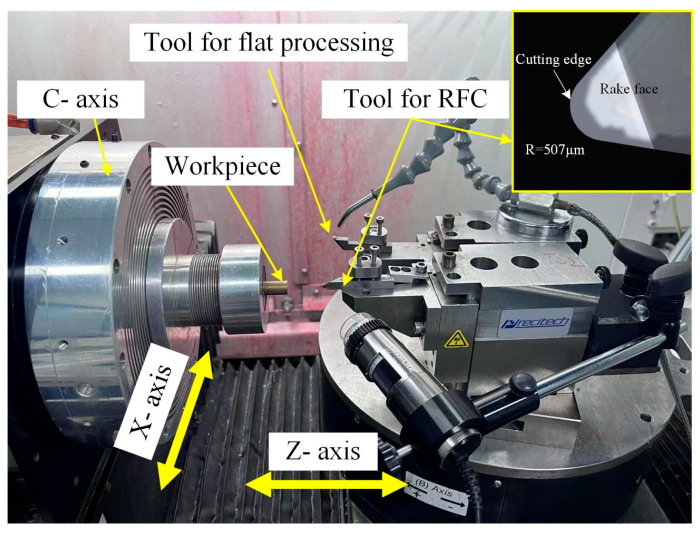
The setup of the experiment.

A high-precision machining platform is essential for achieving high-quality MLA fabrication. In this study, the RPC experiments for MLA mold were conducted on a four-axis ultraprecision machining platform (Nanoform X, Ametek Precitech, Inc., Keene, NH, USA), as shown in Figure 5. To calibrate the tool position precisely, a tool-setting experiment was first performed on the surface of a copper rod. The workpiece was mounted on the C-axis using a vacuum chuck and secured by two precision tool holders that allowed manual adjustment along the Y-axis. The entire assembly was then installed on the B-axis to complete the setup. Two diamond tools were employed: one for surface flattening to ensure workpiece levelness, and the other for RPC to fabricate the MLA mold. The RPC tool featured a diamond tool with a nose radius of 507 μm. A magnified image of the tool, captured using a laser confocal microscope, is presented in Figure 5. For the mold material, a nickel-phosphorus (Ni-P) alloy was selected due to its excellent mechanical properties and suitability for nanoscale precision machining [24]. Based on the tool-setting error control method described in Section 2.3 and the machining capabilities of the RPC process, a 6 × 6 square-arrayed MLA was designed and fabricated to evaluate the machining quality. The curvature radius of the concave spherical lenses was matched to that of the cutting tool, and the pitch between adjacent units was set to 100 μm. Detailed experimental parameters for the MLA mold fabrication are listed in Table 1. Following the machining parameters, the MLA mold was fabricated using the RPC method.

### 3.2. Precision Glass Molding for MLA Optics

**Figure 6:**
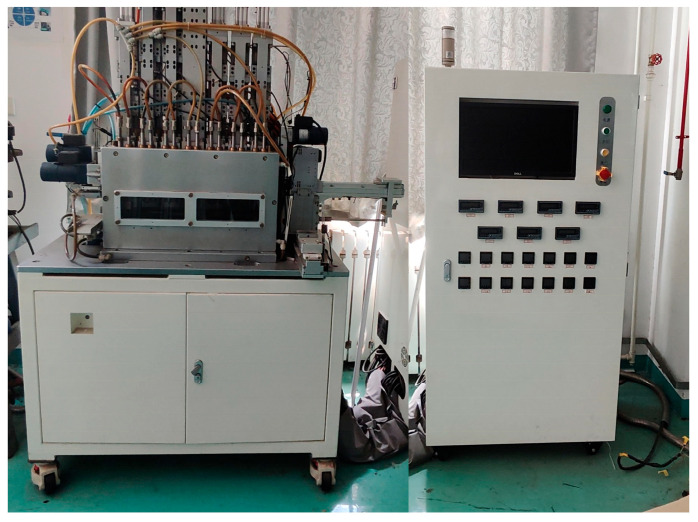
Seven-station precision glass molding machine.

To evaluate the optical imaging performance of the MLA, the mold structure was replicated onto a glass substrate via PGM technology. The PGM experiments were conducted on a self-developed precision molding system, as shown in Figure 6, with K-PG375 glass selected as the replication material. Since the MLA mold features a concave surface, initial contact during the PGM process occurs at the planar regions between the mold and the glass preform, which leads to air trapping. As a result, repeated PGM experiments were required to ensure complete replication, and the replication was considered fully achieved when the fabricated microlens structures exhibited smooth and continuous transitions without any abrupt changes. The processing conditions for a single PGM cycle are summarized in Table 2. All experiments were performed in a nitrogen environment to extend the service life of the mold.

## 4. Results and Discussion

### 4.1. Quantification of Tool Setting Errors

The tool-setting errors *Δx* and *Δy* in the X and Y directions have a significant impact on the precision of machined structures. Based on prior observations, when *Δx* is relatively small, toolpath overlap and chip accumulation are likely to occur, reducing measurement accuracy. To mitigate such interference, the X-direction error was intentionally amplified by offsetting the tool position by 100 μm in the positive X-direction from the origin. By measuring the side length *l*_1_ of the isosceles right triangle formed by the endpoints of three toolpaths, and applying Equation (11), the distance *Δx*′ between the tool’s final position and the rotational center of the C-axis was calculated. The actual tool-setting error *Δx* in the X-direction was determined as the difference between *Δx*′ and 100 μm offset.

**Figure 7:**
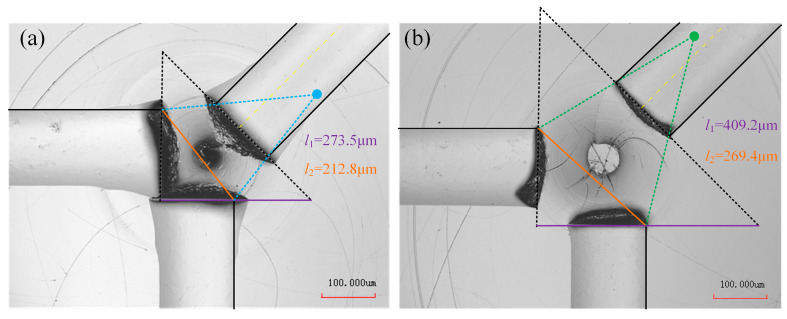
Results under different tool-setting errors: (**a**) type1; (**b**) type2.

**Figure 8:**
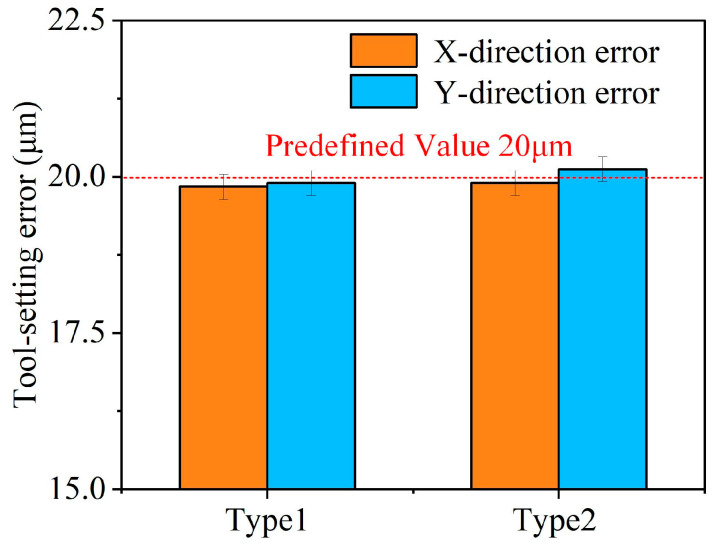
The tool-setting errors in the experiment.

Similarly, the Y-direction tool-setting error *Δy* was obtained by analyzing the positional relationship of toolpath intersections. Its precise value was calculated by substituting the measured *l*_2_ into Equations (12) and (13). To verify the accuracy of this error characterization method, a microscope with a measurement precision of ±0.08 μm was employed. As shown in Figure 7, two sets of tool-setting errors were pre-configured for validation: Type 1 with *Δx* = −20 μm, *Δy* = 20 μm; and Type 2 with *Δx* = 20 μm, *Δy* = −20 μm. By measuring the lengths of *l*_1_ and *l*_2_ and applying the relevant equations, the calculated values of *Δx* and *Δy* were obtained. The experimental results of |*Δx*| and |*Δy*|, shown in Figure 8, confirm that the calculated tool-setting errors deviate by less than 0.2 μm, which satisfies the general requirement of 1 μm accuracy for practical ultraprecision machining applications.

### 4.2. Evaluation of MLA Accuracy

**Figure 9:**
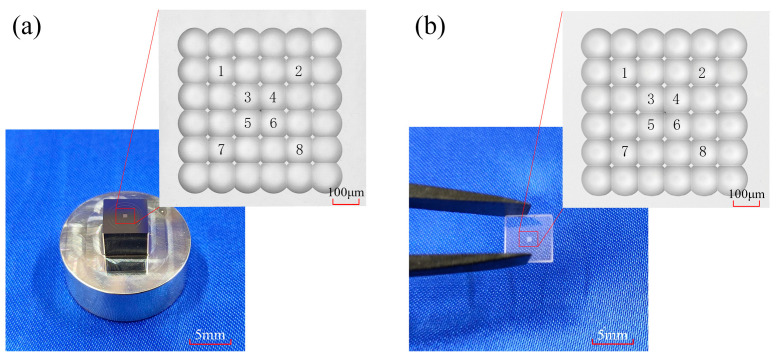
The image of MLA: (**a**) mold; (**b**) lenses.

High-resolution surface characterization of MLA mold and replicated lenses was performed using laser scanning confocal microscopy (VK-X100, Keyence, Osaka, Japan), as shown in Figure 9a,b. The observed surfaces exhibit excellent quality and overall uniformity, with no visible tooling marks, scratches, trapped air, or structural defects. The transition area between adjacent unit displays sharp boundaries and well-defined apex geometries, maintaining high consistency across the entire observation field. These results clearly demonstrate that the RPC method enables precise control over the surface morphology of MLA, fulfilling the requirements for high-precision fabrication.

**Figure 10:**
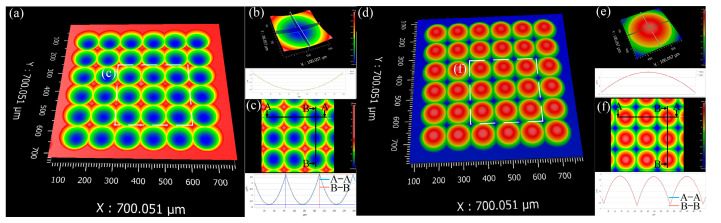
Morphology of MLA mold and lenses: (**a**) 3D topography of mold; (**b**) microlens unit of mold; (**c**) cross-sectional profile of multi-unit in mold; (**d**) 3D topography of lenses; (**e**) microlens unit of lenses; (**f**) cross-sectional profile of multi-unit in lenses.

**Figure 11:**
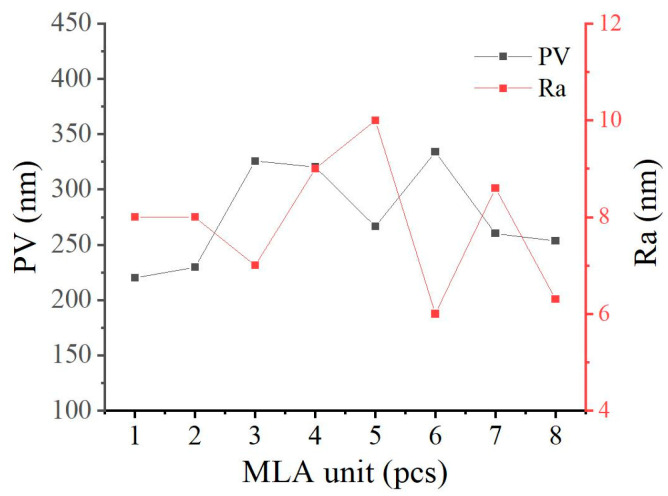
PV and Ra values of the MLA lenses.

The 3D surface morphology of the MLA mold and its replicated lenses was characterized using a white light interferometer (Taylor Hobson, CCI, Leicester, UK), as illustrated in Figure 10. Figure 10a,d display the overall 3D topography of the mold and the replicated lenses, respectively. Figure 10b,e provide detailed views of the 3D morphology and cross-sectional profiles of individual microlens units. Figure 10c,f display the cross-sectional profiles of multiple units within the MLA mold and the replicated lenses, respectively. The results demonstrate that the replicated MLA lenses exhibit high structural uniformity, with only slight variations in depth, likely caused by thermal expansion and contraction effects during the molding process. To further quantify the machining accuracy, a statistical analysis was conducted on the peak-to-valley (PV) and surface roughness (Ra) of four central microlens units and four peripheral units, as illustrated in Figure 11. The measured PV values range from 239 nm to 354 nm, while the surface Ra values range from 5 nm to 11 nm, indicating that the fabricated structures meet the standards for optical-grade quality.

Compared to conventional machining methods, the RPC approach offers significant technical advantages in the fabrication of MLA. By employing tool-sweep-based material removal and optimizing the distribution of toolpath control points, it effectively reduces the machining complexity associated with the dense control-point configurations required in traditional techniques. This method not only simplifies the fabrication process but also improves dimensional precision and structural uniformity, providing a promising technical pathway for the high-quality and scalable fabrication of MLA lenses.

### 4.3. Test of MLA Optical Imaging Performance

**Figure 12:**
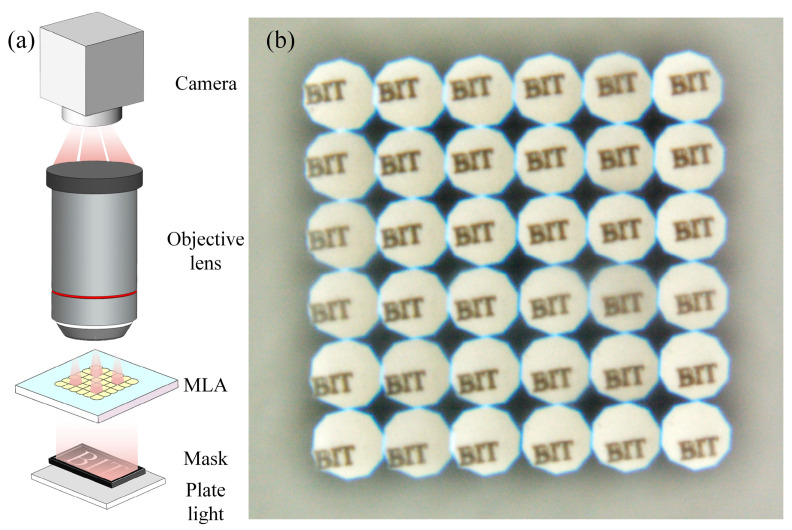
Imaging performance test of MLA: (**a**) schematic of the experimental setup; (**b**) lenses projection image using the ‘BIT’ target.

To assess the practical imaging performance of the fabricated MLA, a series of imaging experiments were conducted. The tests were carried out under white LED illumination using the fabricated MLA lenses, as shown in Figure 12a. The imaging target comprised the uppercase letters “BIT,” laser-ablated onto a transparent glass plate, serving as a standardized resolution test pattern. As depicted in Figure 12b, the recorded images clearly demonstrate the optical uniformity of the MLA, with each microlens unit forming a well-defined and distinct image on the camera sensor. No optical aberrations related to lenses defects were observed across the array, and the imaged features exhibited high consistency in size and shape across the entire observation plane. These results demonstrate that the microlens units exhibit highly uniform focal lengths and aperture sizes, highlighting the accuracy of the structural design and the consistency of the fabrication process.

## 5. Conclusions

This study addresses the challenges of high-precision MLA fabrication by introducing an innovative machining approach based on RPC. The proposed technique offers notable advantages, including reduced motion error, high structural uniformity, and excellent surface quality. By systematically optimizing tool-setting error compensation and toolpath design, the RPC method enables the reliable production of high-quality MLA. The key conclusions are summarized as follows:

(1) An RPC based machining method was developed and implemented on a four-axis ultraprecision machining platform for MLA mold fabrication. By optimizing toolpath control points and dynamically adjusting cutting parameters, significant improvements in surface finish and structural consistency were achieved.

(2) A precise tool-setting error characterization technique was established, enabling accurate alignment of the diamond tool. Tool-setting errors in both X and Y directions were effectively controlled within 1 μm, greatly enhancing machining accuracy and laying the foundation for consistent MLA fabrication.

(3) Using the proposed RPC method, a 6 × 6 MLA lenses with a curvature radius of 507 μm was successfully fabricated. The replicated lenses, produced via PGM, exhibited excellent uniformity and aberration-free optical performance. PV values were maintained below 354 nm, confirming the practical viability of the RPC method.

(4) While the RPC method demonstrates clear advantages in terms of precision, efficiency, and scalability, the present study mainly validates its performance on a 6 × 6 spherical MLA. Its applicability is still constrained by the available tool geometry, which limits the direct fabrication of more complex aspherical profiles. Future work will focus on extending the method to larger-array MLA molds, enabling compatibility with other optical substrate materials, and further enhancing the tool and process design to accommodate more complex surface geometries for broader optical applications.

## Figures and Tables

**Table 1:** Experimental conditions for MLA rotary profile-cutting.

Cutting Parameter	Value
Cutting velocity *v* (mm/min)	3.5
C-axis rotation speed (deg/min)	100
Depth of finish cutting (nm)	30, 60, 120, 240
Tool nose radius *R* (μm)	507
Tool clearance angle (°)	15
Rake angle (°)	0
Machining time (min)	25

**Table 2:** Process parameters in a PGM cycle.

Stage	Temperature (°C)	Heating/Cooling Rate (°C/s)	Force (N)
Rapid heating	25–310	3	0
Slow heating1	310–355	0.3	0
Slow heating2	355–375	0.2	0
Temperature holding & Pressurization	375	0	518
Annealing & Pressure reduction	375–340	0.2	50
Slow cooling	340–300	0.5	50
Rapid cooling	300–25	3	0

## Data Availability

The authors confirm that the data supporting the findings of this study are available within the article.
